# Supplementary material for: Candida albicans pathways that protect against organic peroxides and lipid peroxidation
Source: PLoS Genet. 2024 Oct 21;20(10):e1011455. doi: 10.1371/journal.pgen.1011455 (PMC11527291; doi:10.1371/journal.pgen.1011455)
Supplement: S1 Table — (PDF) [file pgen.1011455.s001.pdf]

Table S1. Strains used in this study.

**Strain**	**Parent**	**Short Genotype**	**Genotype**
LLF100A	SN152	Prototrophic WT control	*ARG4/arg4Δ LEU2/leu2Δ HIS1/his1Δ URA3/ura3Δ::imm434 IRO1/iro1Δ::imm434*
SN152	SC5314	Parental Strain	*arg4Δ/arg4Δ leu2Δ/leu2Δ his1Δ/his1Δ URA3/ura3Δ::imm434 IRO1/iro1Δ::imm434*
KS83A	SN152	*GPX3-GFP* Single Tag	*GPX3-GFP::CdHIS1 ARG4/arg4Δ LEU2/leu2Δ his1Δ/his1Δ URA3/ura3Δ::imm434 IRO1/iro1Δ::imm434*
KS71A	SN152	*GPX31-GFP* Single Tag	*GPX31-GFP::CdHIS1 ARG4/arg4Δ LEU2/leu2Δ his1Δ/his1Δ URA3/ura3Δ::imm434 IRO1/iro1Δ::imm434*
KS84A	SN152	*GPX32-GFP* Single Tag	*GPX32-GFP::CdHIS1 ARG4/arg4Δ LEU2/leu2Δ his1Δ/his1Δ URA3/ura3Δ::imm434 IRO1/iro1Δ::imm434*
KS22A	SN152	*GPX3-GFP* Double Tag	*GPX3-GFP::CdHIS1 GPX3-GFP::CmLeu2 ARG4/arg4Δ leu2Δ/leu2Δ his1Δ/his1Δ URA3/ura3Δ::imm434 IRO1/iro1Δ::imm434*
KS23A	SN152	*GPX31-GFP* Double Tag	*GPX31-GFP::CdHIS1 GPX31-GFP::CmLEU2 ARG4/arg4Δ leu2Δ/leu2Δ his1Δ/his1Δ URA3/ura3Δ::imm434 IRO1/iro1Δ::imm434*
KS24A	SN152	*GPX32-GFP* Double Tag	*GPX32-GFP::CdHIS1 GPX32-GFP::CmLEU2 ARG4/arg4Δ leu2Δ/leu2Δ his1Δ/his1Δ URA3/ura3Δ::imm434 IRO1/iro1Δ::imm434*
KS27A	SN152	*gpx3Δ/Δ*	*gpx3Δ::CdHIS1/gpx3Δ::CdHIS1 ARG4/arg4Δ LEU2/leu2Δ his1Δ/his1Δ URA3/ura3Δ::imm434 IRO1/iro1Δ::imm434*
KS98B	SN152	*gpx31,32,33Δ/Δ* (*GPX3* only)	*gpx31-32-33Δ::CdHIS1/gpx31-32-33Δ::CdHIS1 ARG4/arg4Δ LEU2/leu2Δ his1Δ/his1Δ URA3/ura3Δ::imm434 IRO1/iro1Δ::imm434*
KS49A	SN152	*GPX31* only	*gpx3Δ::CdHIS1/gpx3Δ::CdHIS1 gpx33Δ::CmLEU2/gpx33Δ::CmLEU2 gpx32Δ::CdARG4/gpx32Δ::CdARG4 arg4Δ/arg4Δ leu2Δ/leu2Δ his1Δ/his1Δ URA3/ura3Δ::imm434 IRO1/iro1Δ::imm434*
KS51A	SN152	*GPX32* only	*gpx3Δ::CdHIS1/gpx3Δ::CdHIS1 gpx31-33Δ::CmLEU2/gpx31-33Δ::CmLEU2 ARG4/arg4Δ leu2Δ/leu2Δ his1Δ/his1Δ URA3/ura3Δ::imm434 IRO1/iro1Δ::imm434*
KS50A	SN152	*GPX33* only	*gpx3Δ::CdHIS1/gpx3Δ::CdHIS1 gpx31-32Δ::CmLEU2/gpx31-32::CmLEU2 ARG4/arg4Δ leu2Δ/leu2Δ his1Δ/his1Δ URA3/ura3Δ::imm434 IRO1/iro1Δ::imm434*
KS39A	SN152	*gpxΔ/Δ/Δ/Δ*	*gpx3Δ::CdHIS1/gpx3Δ::CdHIS1 gpx31-32-33Δ::CmLEU2/gpx31-32-33Δ::CmLEU2 ARG4/arg4Δ leu2Δ/leu2Δ his1Δ/his1Δ URA3/ura3Δ::imm434 IRO1/iro1Δ::imm434*
KS57A	SN152	*GPX* Comp.	*gpx3Δ::CdHIS1/gpx3Δ::CdHIS1 gpx31-32-33Δ::CmLEU2/gpx31-32-33Δ::CmLEU2 GPX3-31-32-33::NAT::NEUT5L ARG4/arg4Δ leu2Δ/leu2Δ his1Δ/his1Δ URA3/ura3Δ::imm434 IRO1/iro1Δ::imm434*
MT505-A	SC5314	*cat1Δ/Δ*	*cat1Δ::frt/cat1Δ::frt*
KS64A	SN152	*cap1Δ/Δ*	*cap1Δ::CmLEU2/cap1Δ::CdHIS1 ARG4/arg4Δ leu2Δ/leu2Δ his1Δ/his1Δ URA3/ura3Δ::imm434 IRO1/iro1Δ::imm434*

KS89A	SN152	*CAP1* Comp.	*cap1Δ::CmLEU2/cap1Δ::CdHIS1 CAP1::NAT::NEUT5L ARG4/arg4Δ leu2Δ/leu2Δ his1Δ/his1Δ URA3/ura3Δ::imm434 IRO1/iro1Δ::imm434*
KS79C	SN152	*gpxΔ/Δ/Δ/Δ+cap1Δ/Δ*	*gpx3Δ::CdHIS1/gpx3Δ::CdHIS gpx31-32-33Δ::CmLEU2/gpx31-32-33ΔCmLEU2 cap1Δ::CdARG4/cap1Δ::CdARG4 arg4Δ/arg4Δ leu2Δ/leu2Δ his1Δ/his1Δ URA3/ura3Δ::imm434 IRO1/iro1Δ::imm434*
KS78B	SN152	*cap1Δ/Δ+GPX3-GFP*	*cap1Δ::CmLEU2/cap1Δ::CdHIS1 GPX3-GFP::CdARG4 arg4Δ/arg4Δ leu2Δ/leu2Δ his1Δ/his1Δ URA3/ura3Δ::imm434 IRO1/iro1Δ::imm434*
KS73A	SN152	*cap1Δ/Δ+GPX31-GFP*	*cap1Δ::CmLEU2/cap1Δ::CdHIS1 GPX31-GFP::CdARG4 arg4Δ/arg4Δ leu2Δ/leu2Δ his1Δ/his1Δ URA3/ura3Δ::imm434 IRO1/iro1Δ::imm434*
KS74A	SN152	*cap1Δ/Δ+GPX32-GFP*	*cap1Δ::CmLEU2/cap1Δ::CdHIS1 GPX32-GFP::CdARG4 arg4Δ/arg4Δ leu2Δ/leu2Δ his1Δ/his1Δ URA3/ura3Δ::imm434 IRO1/iro1Δ::imm434*
YLD240-8-2	SN152	*trx1Δ/Δ*	*trx1Δ::LEU2/trx1Δ::LEU2 ARG4/arg4Δ HIS1Δ/his1Δ leu2Δ/leu2Δ URA3/ura3Δ::imm434 IRO1/iro1Δ::imm434*
KS87A	SN152	*glr1Δ/Δ*	*glr1Δ::CdARG4/glr1Δ::CdARG4 arg4Δ/arg4Δ LEU2/leu2Δ HIS1/his1Δ URA3/ura3Δ::imm434 IRO1/iro1Δ::imm434*
KS91A	SN152	*GLR1* Comp.	*glr1Δ::CdARG4/glr1Δ::CdARG4 GLR1::NAT::NEUT5L arg4Δ/arg4Δ LEU2/leu2Δ HIS1/his1Δ URA3/ura3Δ::imm434 IRO1/iro1Δ::imm434*
KS88A	SN152	*trr1Δ* (Heterozygous)	*trr1Δ::CdARG4 arg4Δ/arg4Δ LEU2/leu2Δ HIS1/his1Δ URA3/ura3Δ::imm434 IRO1/iro1Δ::imm434*
KS95A	SN152	*tsa1Δ/Δ+ tsa1bΔ/Δ*	*tsa1Δ::CdARG4/tsa1Δ::CdARG4 tsa1bΔ::CdARG4/tsa1bΔ::CdARG4 arg4Δ/arg4Δ LEU2/leu2Δ HIS1/his1Δ URA3/ura3Δ::imm434 IRO1/iro1Δ::imm434*
YLD184-3	SN152	*hog1Δ/Δ*	*hog1Δ::CdHIS1/hog1Δ::CmLEU2 ARG4/arg4Δ LEU2/leu2Δ his1Δ/his1Δ URA3/ura3Δ::imm434 IRO1/iro1Δ::imm434*
YLD197-1	SN152	*pbs2Δ/Δ*	*pbs2Δ::CmLEU2/pbs2Δ::CdHIS1 ARG4/arg4Δ LEU2/leu2Δ his1Δ/his1Δ URA3/ura3Δ::imm434 IRO1/iro1Δ::imm434*
YLD185-7	SN152	*ssk2Δ/Δ*	*ssk2Δ::CmLEU2/ssk2Δ::CdHIS1 ARG4/arg4Δ LEU2/leu2Δ his1Δ/his1Δ URA3/ura3Δ::imm434 IRO1/iro1Δ::imm434*
YHXW4	BWP17	*SUR7-GFP*	*ura3Δ::λimm434/ura3Δ::λimm434 his1::hisG/his1::hisG arg4::hisG/arg4::hisG SUR7-GFPγ::URA3*
Homann, X2	SN152	*cap1Δ/Δ*	*cap1Δ::CmLEU2/cap1Δ::CdHIS1 arg4Δ/arg4Δ leu2Δ/leu2Δ his1Δ/his1Δ URA3/ura3Δ::imm434 IRO1/iro1Δ::imm434*
Homann, Y2	SN152	*cap1Δ/Δ*	*cap1Δ::CmLEU2/cap1Δ::CdHIS1 arg4Δ/arg4Δ leu2Δ/leu2Δ his1Δ/his1Δ URA3/ura3Δ::imm434 IRO1/iro1Δ::imm434*
Noble, Plate 3	SN152	*cap1Δ/Δ*	*cap1Δ::CmLEU2/cap1Δ::CdHIS1 arg4Δ/arg4Δ leu2Δ/leu2Δ his1Δ/his1Δ URA3/ura3Δ::imm434 IRO1/iro1Δ::imm434*
Noble, Plate 11	SN152	*cap1Δ/Δ*	*cap1Δ::CmLEU2/cap1Δ::CdHIS1 arg4Δ/arg4Δ leu2Δ/leu2Δ his1Δ/his1Δ URA3/ura3Δ::imm434 IRO1/iro1Δ::imm434*
