# Supplementary material for: Candida albicans pathways that protect against organic peroxides and lipid peroxidation
Source: PLoS Genet. 2024 Oct 21;20(10):e1011455. doi: 10.1371/journal.pgen.1011455 (PMC11527291; doi:10.1371/journal.pgen.1011455)
Supplement: S3 Table — (PDF) [file pgen.1011455.s003.pdf]

Table S3. LNA Spot Assay Agar Solutions

Plate	0mM (Control)	5mM OA	2mM LNA	5mM LNA
LNA	-------------------	-----------------	42μL	106μL
OA	-------------------	79μL	-----------------	------------------
10% tergitol in SD+uri	5mL	5mL	5mL	5mL
DMSO	106μL	27μL	64μL	------------------
SC+uri agar	44.9mL	44.9mL	44.9mL	44.9mL
