# Supplementary material for: Candida albicans pathways that protect against organic peroxides and lipid peroxidation
Source: PLoS Genet. 2024 Oct 21;20(10):e1011455. doi: 10.1371/journal.pgen.1011455 (PMC11527291; doi:10.1371/journal.pgen.1011455)
Supplement: S4 Table — (PDF) [file pgen.1011455.s004.pdf]

Table S4. LNA Dose Response Assay Solutions

M LNA	10% tergitol in SD+uri	SD+uri	LNA	1:10 LNA dilution in DMSO	DMSO
1.5	5μL	22μL	23μL	----------------------------------	----------
0.75	5μL	22μL	11.4μL	----------------------------------	11.6μL
0.38	5μL	22μL	5.8μL	----------------------------------	17.2μL
0.19	5μL	22μL	2.9μL	----------------------------------	20.1μL
0.09	5μL	22μL	---------	14μL	9μL
0.05	5μL	22μL	---------	8μL	15μL
0.02	5μL	22μL	---------	3μL	20μL
0.01	5μL	22μL	---------	1.5μL	21.5μL
0	5μL	22 μL	---------	----------------------------------	23μL
M OA	10% tergitol in SD+uri	SD+uri	OA	----------------------------------	----------
1.5	5μL	21.2μL	23.8μL	----------------------------------	----------
