# Supplementary figures and images for: Candida albicans pathways that protect against organic peroxides and lipid peroxidation
Source: PLoS Genet. 2024 Oct 21;20(10):e1011455. doi: 10.1371/journal.pgen.1011455 (PMC11527291; doi:10.1371/journal.pgen.1011455)

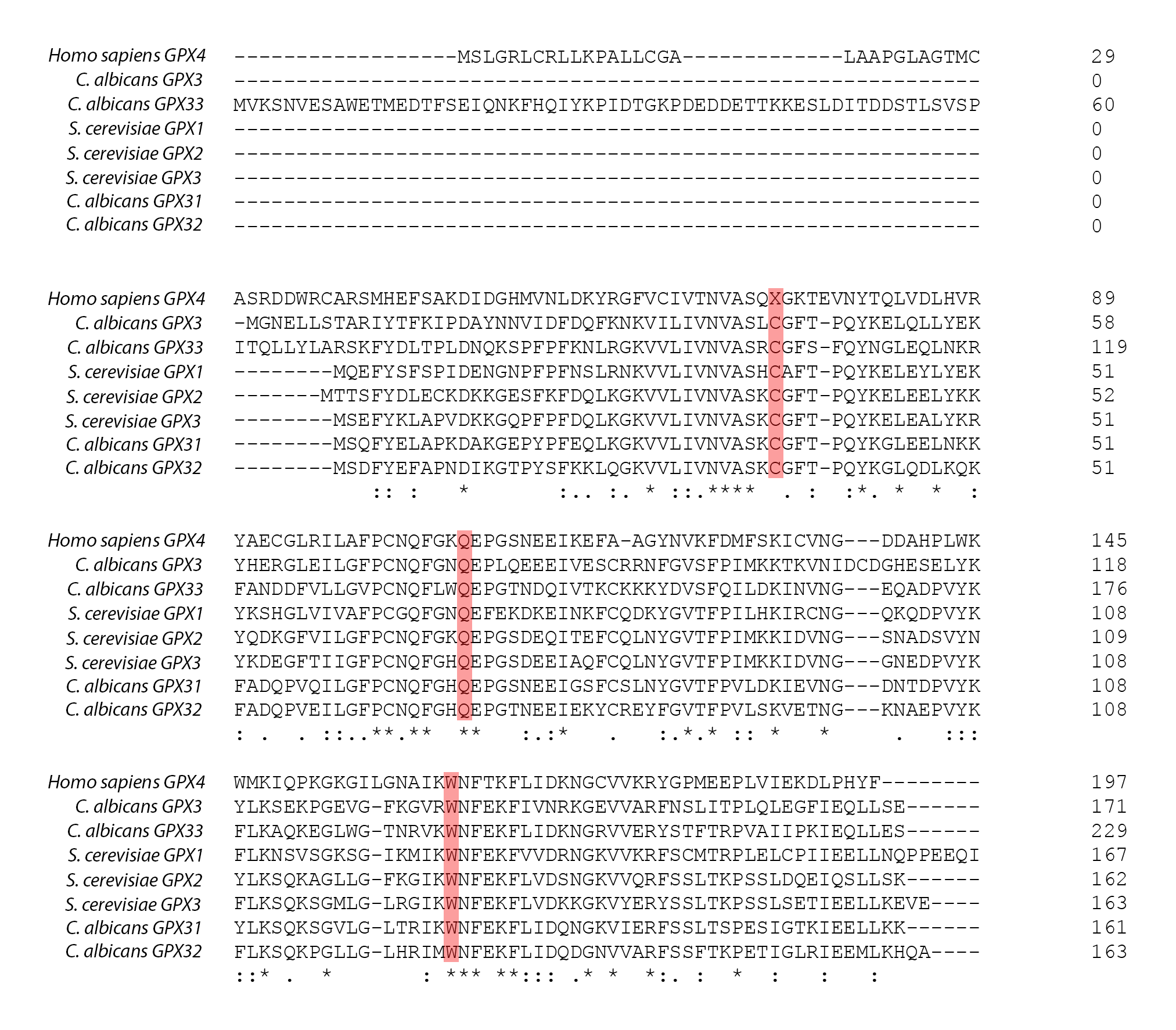

Supplement: S1 Fig — Sequence alignment for the four C. albicans Gpx proteins, the three S. cerevisiae Gpx proteins, and the human Gpx4 protein. Residues highlighted in red mark the Cys, Gln, and Trp catalytic triad. Note that for Homo sapiens Gpx4, the catalytic triad contains a selenocysteine (“X”) at residue 73 and not a Cys. Asterisks indicate completely conserved residues, a single dot indicates partially conserved residues, and a colon denotes residues with similar properties. Alignment was created using Clustal software (https://www.ebi.ac.uk/jdispatcher/msa/clustalo). (TIF) [file pgen.1011455.s009.tif]

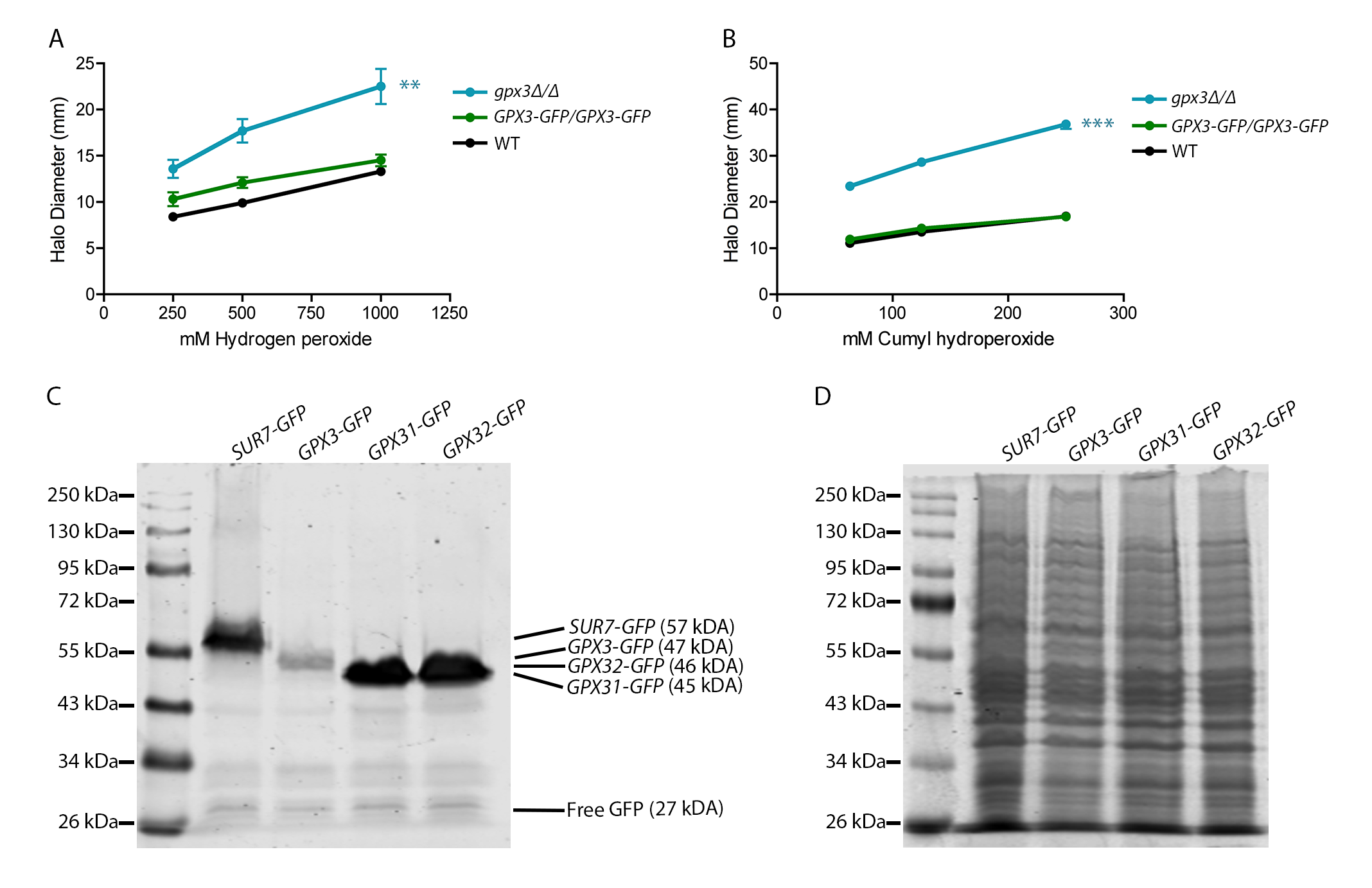

Supplement: S2 Fig — To determine if GFP tagged Gpx3 is functional, a GPX3-GFP strain tagged on both GPX3 alleles was compared to WT and gpx3Δ/Δ strains to assess any increased sensitivity to oxidative stress. Disk diffusion halo assays were conducted with (A) H2O2 or (B) CHP at 30°C with 3–4 independent assays carried out per strain. Asterisks indicate strains with statistically significant differences in values at all concentrations when compared to WT. Strains with no asterisks had no significant differences from WT. The GPX3-GFP strain was tested because the gpx3Δ/Δ mutant had a measurable phenotype. (C) Western blot analysis indicating that the Gpx-GFP fusion proteins were full-length and that there was little or no evidence of a free GFP tag that had been proteolytically cleaved. A SUR7-GFP strain was used as a positive control. Strains with both GPX alleles tagged with GFP were used. (D) Coomassie stained gel to compare relative protein levels for samples used in (C). The Western blots and respective Coomassie stained gels were completed in triplicate. Statistical analysis for halo assays used one-way ANOVA with Tukey’s multiple comparison test. * p<0.05, ** p<0.01, and *** p<0.001. (TIF) [file pgen.1011455.s010.tif]

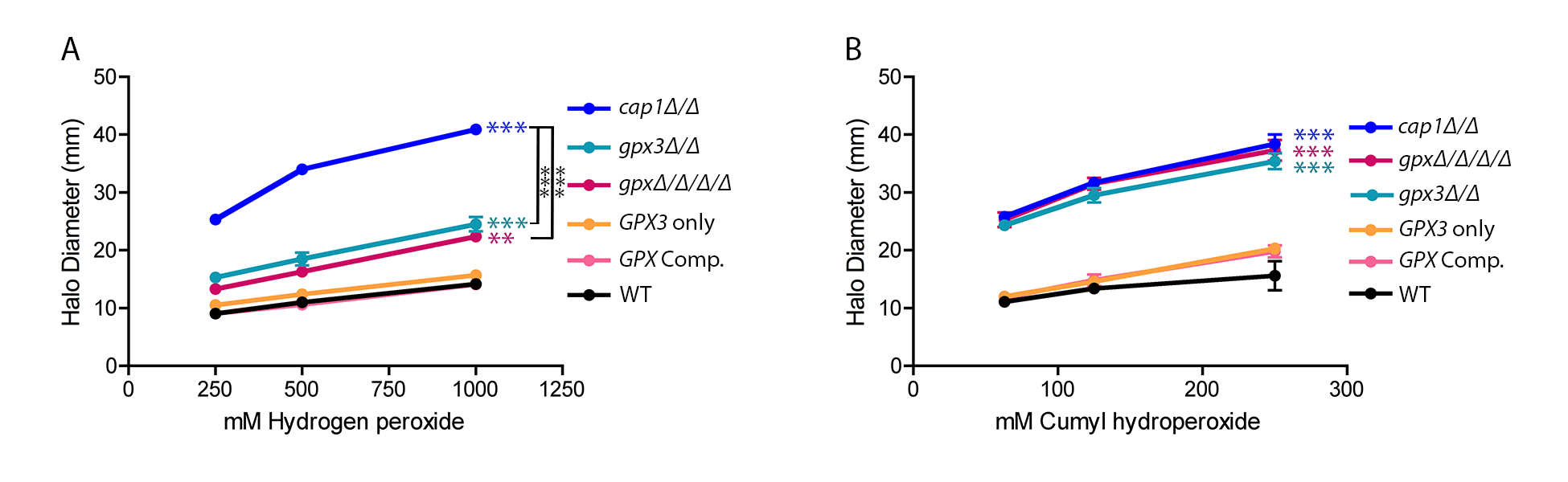

Supplement: S3 Fig — To determine oxidative stress sensitivity of key mutant strains at physiological temperature, disk diffusion halo assays were conducted with (A) H2O2 or (B) CHP at 37°C. The diameter of the zone of inhibition was calculated after 48 h. The results represent 3–4 independent assays. Colored asterisks indicate strains with significantly different values at all concentrations when compared to WT. Strains with no asterisks had no significant differences from WT. Brackets with black asterisks indicate select strains of interest with significant differences between all values. Statistical analysis for halo assays used one-way ANOVA with Tukey’s multiple comparison test. * p<0.05, ** p<0.01, and *** p<0.001. (TIF) [file pgen.1011455.s011.tif]

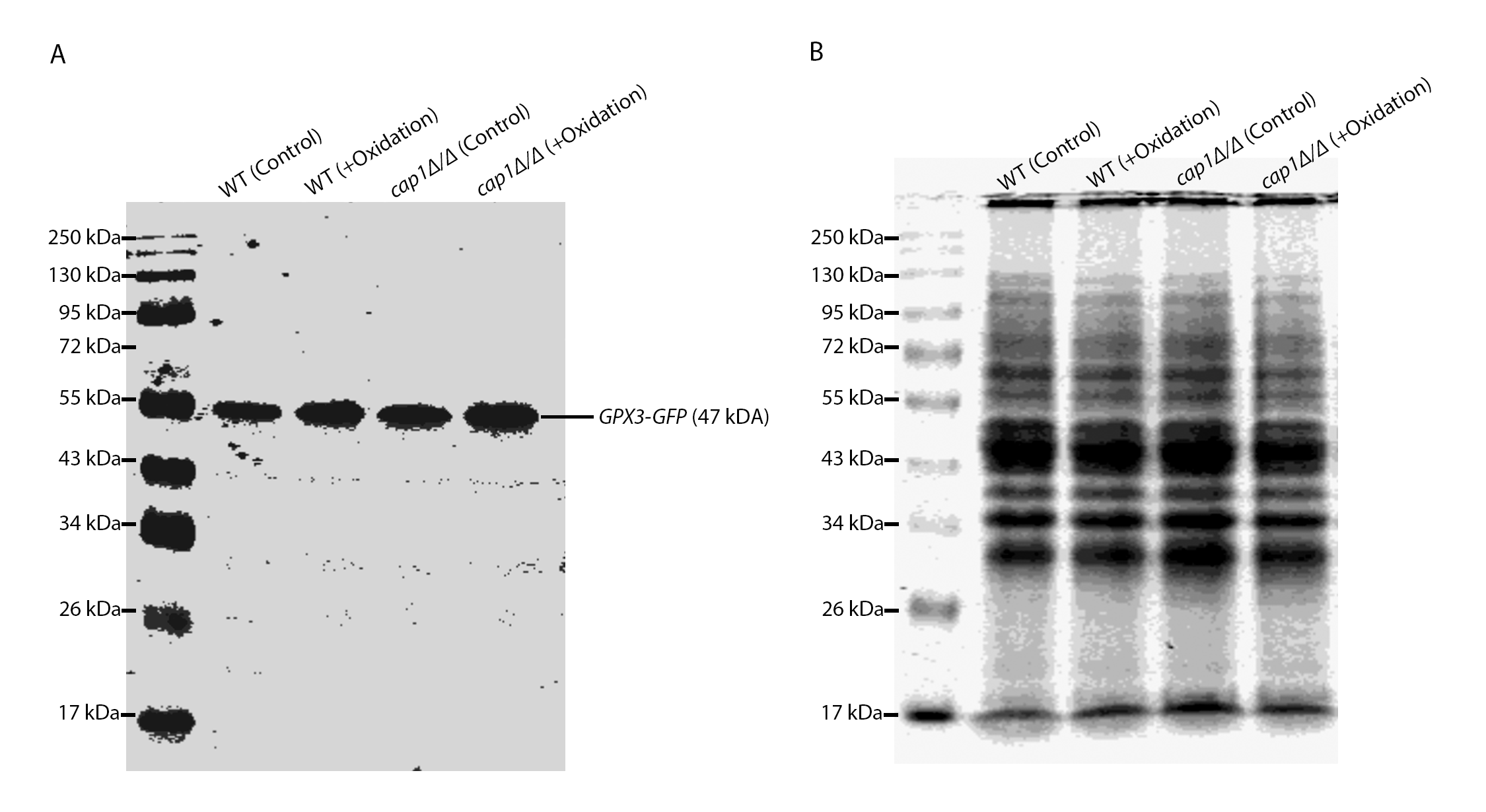

Supplement: S4 Fig — Western blots were conducted for WT and cap1Δ/Δ strains that each carried a single copy of GPX3-GFP. (A) Western blot showing Gpx3-GFP levels in WT and cap1Δ/Δ strains with and without exposure to 0.3 mM t-BHP for 30 min. (B) Corresponding Coomassie gel showing the total protein levels of the same samples in (A). These are representative images from 4 independent assays. (TIF) [file pgen.1011455.s012.tif]

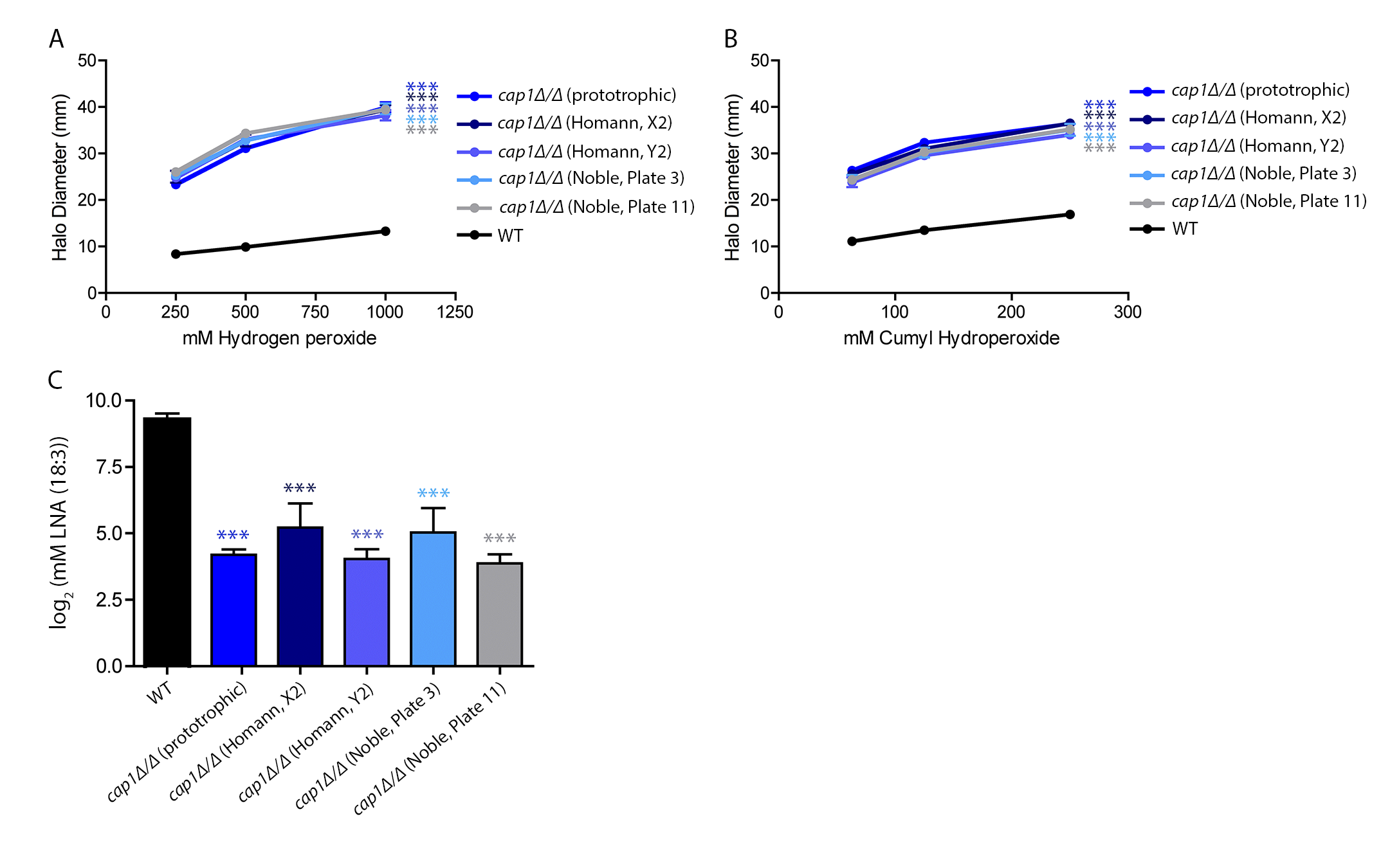

Supplement: S5 Fig — To determine the oxidative stress sensitivity of various cap1Δ/Δ strains, disk diffusion halo assays were conducted with (A) H2O2 or (B) CHP. Plates were incubated at 30°C and the diameter of the zone of inhibition was measured after 48 h. The results represent 3–4 independent assays. (C) Quantitative assay for sensitivity to LNA (18:3). All assays compared the sensitivity of WT and five cap1Δ/Δ strains: the prototrophic cap1Δ/Δ strain, the two cap1Δ/Δ strains from the Homann collection, and the two cap1Δ/Δ strains from the Noble collection. Assays for each strain were carried out a minimum of 3 independent times. Asterisks indicate strains with significantly different values at all concentrations when compared to WT. No significant differences in sensitivity were found between the five cap1Δ/Δ strains in any of the assays. Statistical analysis for assays used one-way ANOVA with Tukey’s multiple comparison test. * p<0.05, ** p<0.01, and *** p<0.001. (TIF) [file pgen.1011455.s013.tif]

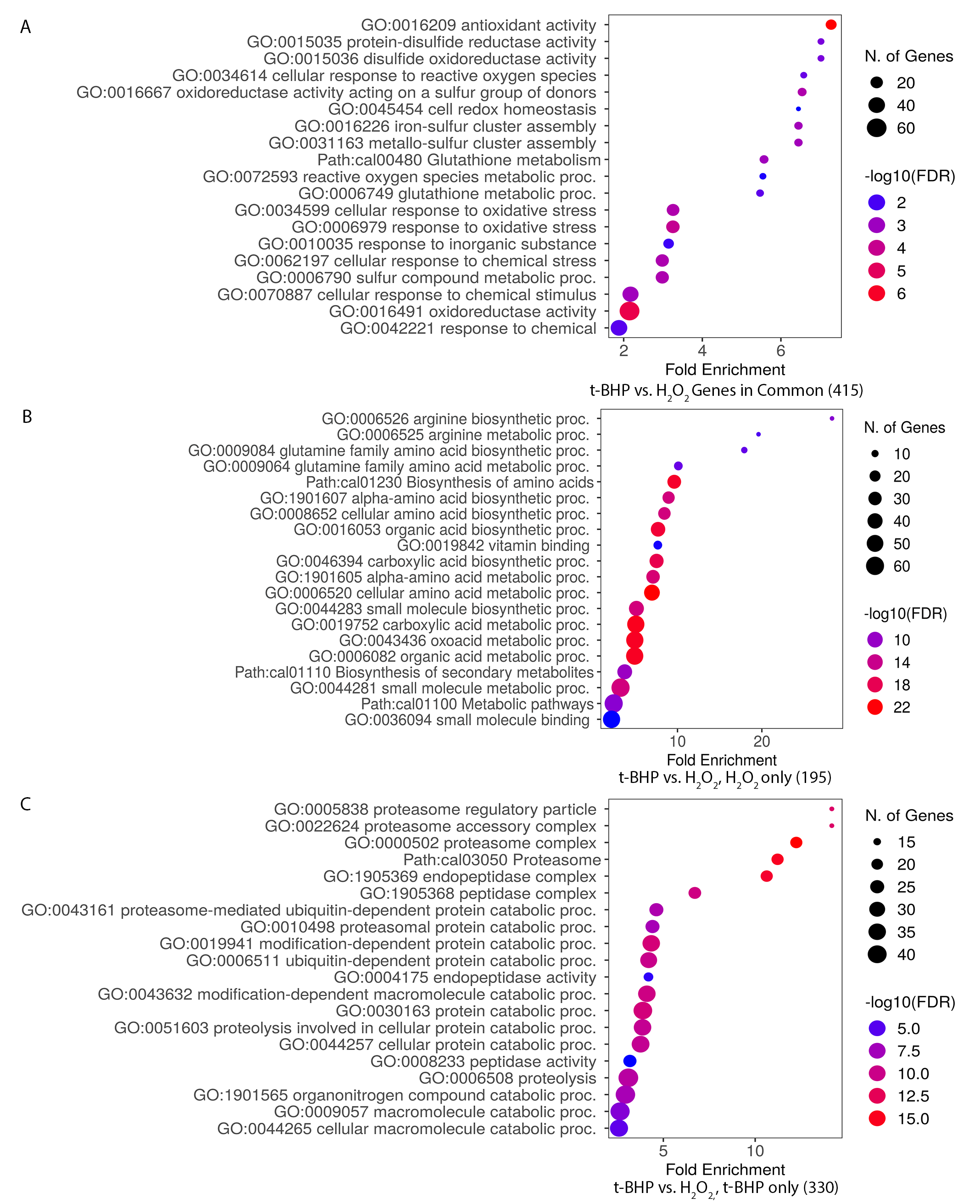

Supplement: S6 Fig — GO terms analysis for comparing the t-BHP and H2O2 transcriptional responses. GO terms were determined for transcriptional responses that had a log2(Fold Change) value of ≥ 1.00, which is equivalent to a ≥ 2-fold transcriptional upregulation when compared to control samples. (A) GO terms for ≥ 2-fold upregulated genes in common between the H2O2 and t-BHP treated samples. (B) GO terms for genes upregulated ≥ 2-fold upon exposure to H2O2, but not t-BHP. (C) GO terms for genes upregulated ≥ 2-fold with exposure to t-BHP, but not H2O2. Only transcriptional responses with P-adj. < 0.05 were used in the analysis. (TIF) [file pgen.1011455.s014.tif]

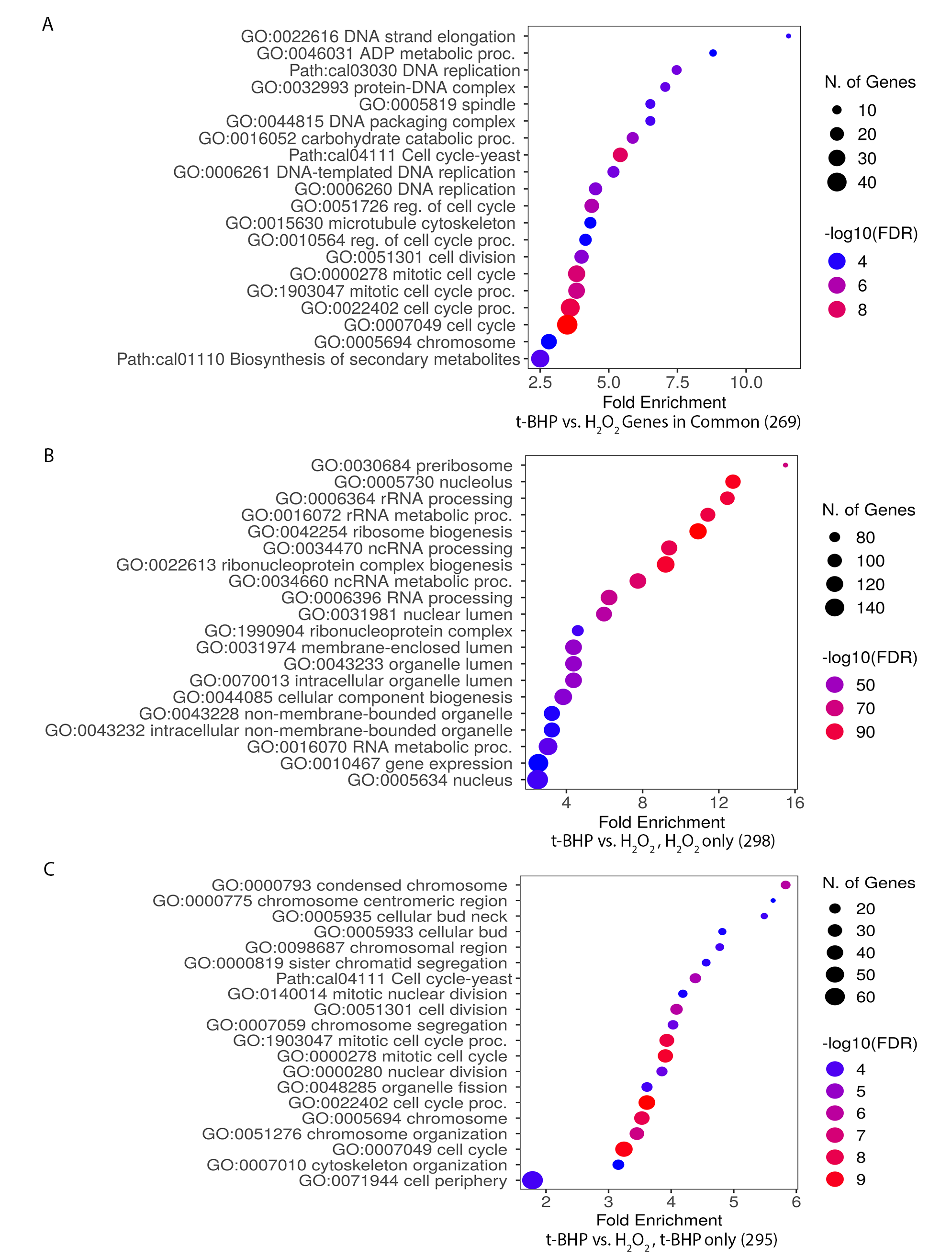

Supplement: S7 Fig — GO terms analysis comparing the t-BHP and H2O2 transcriptional responses. GO terms determined for transcriptional responses that had a log2(Fold Change) value of ≤ -1.00, which is equivalent to a ≥ 2-fold transcriptional downregulation when compared to control samples. (A) GO terms for ≥ 2-fold downregulated genes in common between the H2O2 and t-BHP treated samples. (B) GO terms for genes downregulated ≥ 2-fold upon exposure to H2O2, but not t-BHP. (C) GO terms for genes downregulated ≥ 2-fold with exposure to t-BHP, but not H2O2. Only transcriptional responses with P-adj. < 0.05 were used in the analysis. (TIF) [file pgen.1011455.s015.tif]
